# Patient Perceptions of Allergic Rhinitis and Quality of Life: *Findings From a Survey Conducted in Europe and the United States*

**DOI:** 10.1097/WOX.0b013e3181865faf

**Published:** 2008-09-15

**Authors:** G Walter Canonica, Joaquim Mullol, André Pradalier, Alain Didier

**Affiliations:** 1 Allergy and Respiratory Diseases Clinic, DIMI, University of Genoa, Genoa, Italy; 2 Rhinology Unit, ENT Department, Hospital Clinic, Barcelona, Catalonia, Spain; 3 Department of Internal Medicine, Allergology Service, Hôpital Louis Mourier, Colombes Cédex, France; 4 Service de Pneumologie et Allergologie, Hôpital Larrey CHU de Toulouse, France

**Keywords:** allergic rhinitis, nonallergic rhinitis, quality of life

## Abstract

**Background:**

Allergic rhinitis (AR) is a common, costly, and troublesome condition, impairing patients' quality of life (QoL), cognitive function, and productivity. Patients with AR report disturbed sleep, fatigue, irritability, and a range of practical problems. However, there is a relative lack of data on how patients with AR perceive their QoL.

**Objective:**

To better understand how patients perceive AR and their attitudes toward this condition (including QoL) and its treatment options.

**Methods:**

An online and telephone survey of 3635 people identified as having outdoor and indoor allergies, urticaria, and/or pet allergies was performed in 6 countries.

**Results:**

The survey confirmed that patients with allergies perceive their symptoms as causing significant disruptions to their daily lives. Respondents were affected for a considerable part of each day, with the most severe symptoms occurring in the morning. The most important desired effect of medication was the restoration of normal breathing, and the most highly rated attributes of the "ideal" AR drug were efficacy, safety, and freedom from undesirable side effects.

**Conclusions:**

The information gathered from allergy sufferers who participated in this survey sheds light on the degree to which people with allergies are affected by their disease and the limitations imposed by associated symptoms.

## 

In clinical trials of allergic rhinitis (AR), quality of life (QoL) is assessed by questionnaires that ask subjects about the effects of AR on various domains of their daily lives, including practical functions, social interactions, emotional well-being, and economic activity. These instruments comprise both generic questionnaires that measure physical, mental, and psychosocial functioning, such as the Medical Outcomes Study 36-Item Short-Form Health Survey,[1,2] and ones that are more disease specific, such as the Rhinoconjunctivitis Quality of Life Questionnaire (RQLQ), Rhinasthma, and the Rhinosinusitis Disability Index [3-5]. The effects of AR on health-related QoL extend to learning, sleep, vitality/alertness, perception of general health, cognitive and emotional functioning, and psychomotor performance [6-8]. Strong, highly significant correlations have been found between clinical AR symptoms and QoL [6]. Such limitations on daily functioning can have negative effects on a person's performance at home, school, or work, which have both a direct and an indirect economic impact on society [9].

An online survey conducted in 2004 to assess QoL in 2002 patients with AR and nasal congestion (or their caregivers) has reinforced these findings [10]. Within the subgroup of working adults (n = 1043), 59% reported that their congestion had affected their job performance. Of the 446 caregivers, 42% said that congestion led to their child's poor productivity in school. Nasal congestion associated with AR also led to feelings of discomfort, frustration, fatigue, irritability, and stress [10]. A Swedish survey of 9538 adolescents with AR and/or asthma found that severe nasal symptoms were associated with lower grades in school [11].

Although the typical clinical symptoms of AR might be considered relatively inconsequential, they are consistently mentioned by patients as having a significant impact on QoL. Thus, there may be a disconnect between clinicians' perceptions of AR as a chronic but nonserious medical condition that causes a limited range of symptoms and patients' perceptions of it as a limiting and disabling presence in their lives. This disconnect may in turn lead to suboptimal treatment.

To address the way health care providers and patients perceive the effects of AR, a multinational survey, "Understanding the Dynamics of Allergy Suffering and Treatment," conducted by the Forbes Consulting Group, was undertaken. The objective was to better understand patients' attitudes and perceptions about the symptoms and effects of AR, treatment approaches, and available therapies. The survey investigated respondents' product or brand choices and what form of therapy they preferred (eg, tablets, nasal sprays). Some methodologic flexibility to define concepts was permitted within each country.

## Methods

A questionnaire was administered in July 2005 to respondents in France, Germany, Italy, Spain, the United Kingdom, and the United States, with the goal of obtaining a minimum of 575 complete responses from each country (3450 total): 500 from people who treated their allergies and 75 from nontreaters. The actual number of complete responses received was 3635. Respondent panels were recruited in a way that the geographic distribution of the panel within each country accurately reflected the population distribution.

The survey was conducted by Internet, except in Spain, where it was conducted by telephone. To ensure uniformity of responses and to control for scale-use bias, scale variables were standardized, setting the mean within each variable and within each observation to zero and the SD to 1. A follow-up interview was conducted by Internet in the United States in November 2005.

Respondents were included only if they had experienced symptoms related to outdoor, indoor, pet, mold, or dust allergies, including AR and urticaria, during the preceding 12 months. People who primarily had food, insect, or medication allergies were excluded.

Survey participants answered questions on the frequency and duration of symptoms, the times of day and the season(s) in which they occurred, and what types of symptoms they experienced. They were given a list of the core allergy symptoms and were asked to note which ones they had: sneezing, itchy/watery eyes, nasal congestion, runny nose, sinus congestion, postnasal drip, sinus pressure, itchy throat, sinus pain, and hives/rash. Patients were also asked to use a list of 9 possible responses to rate the emotional impact of their morning allergy symptoms: more irritable, more fatigued, less motivated, less energetic, hard to concentrate, more frustrated, less alert, more self-conscious, and feelings not affected. There were questions about how disruptive nasal allergies in general (and congestion specifically) were to various aspects of everyday life, including sleep, participation in social and outdoor activities, and mood. Data were weighted to age, sex, and household income distributions. Respondents in the United States were subdivided according to 3 levels of symptom severity (mild, moderate, and severe).

Lifestyle impact was calculated with index scores based on symptoms and lifestyle disruption. The prevalence of specific symptoms was multiplied by symptom severity and was indexed against the overall average to create individual index scores for the various symptoms. Prevalence of lifestyle difficulties was multiplied by disruptiveness and was indexed against the overall average to create individual scores.

## Results

### Demographics

Respondent demographics are listed in Table 1. The percentage of male patients was similar in the 6 countries, but the survey participants' ages and overall health varied among the countries.

**Table 1:** Respondent Demographic

	France, n = 595	**Germany**, n = 676	**Italy**, n = 575	**Spain**, n = 575	**United Kingdom**, n = 615	**United States**, n = 599
Male	48	47	49	49	48	49
sex, %						
Mean	39	39	38	44	42	44
age, yr						
Overall						
health, %						
Excellent	8	3	6	9	8	9
Very good	30	26	30	17	31	39
Good	46	52	46	40	33	35
Fair	14	16	16	29	25	13
Poor	3	4	2	6	4	3

### Incidence

**Figure 1:**
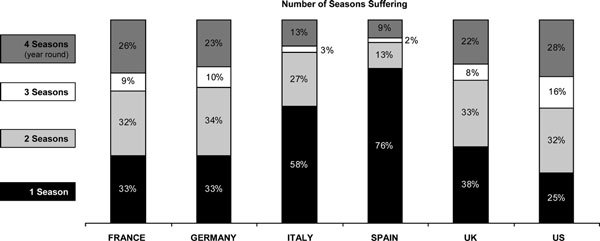
**Number of seasons experiencing allergy symptoms**.

There was considerable intercountry variability with regard to the seasons in which AR was experienced. Forty-four percent of respondents in the United States experienced 3- or 4-season allergies, followed by France (35%), Germany (33%), and the United Kingdom (30%), with much lower incidences reported in Italy (16%) and Spain (11%) (Figure 1).

**Figure 2:**
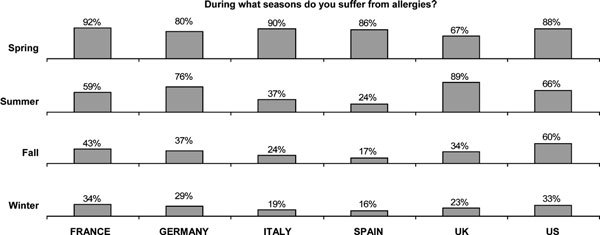
**Time of year when allergy symptoms were experienced**.

In most countries, the highest percentage of respondents experienced allergy symptoms in the spring, led by France (92%). The only exception was in the United Kingdom, where the highest percentage (89%) reported allergies during the summer (Figure 2).

Respondents in the United States reported having the greatest number of days experiencing allergy symptoms per year (102), followed by the United Kingdom (79), Germany (68), France (62), Italy (37), and Spain (33). Survey participants in the United Kingdom reported the highest number of annual days with indoor allergies (160), followed by the United States (129), Germany (103), France (67), Italy (59), and Spain (36).

The Allergic Rhinitis and its Impact on Asthma Workshop recommended that the terms seasonal and perennial AR be replaced with intermittent AR (IAR) and persistent AR (PER). Intermittent AR is defined as the presence of rhinitis symptoms for less than 4 days/wk or for less than 4 weeks, whereas in PER, rhinitis symptoms are present for greater than 4 days/wk and for greater than 4 weeks [12]. Table 2 lists the distribution of patients with IAR and PER in the 6 study countries. Spain had the most patients with IAR (70%), whereas both the United Kingdom and the United States had the greatest percentage of respondents with PER (53% and 52%, respectively). This is consistent with the number of reported symptom days.

**Table 2:** Distribution of IAR and PER

AR Type	France	Germany	Italy	Spain	United Kingdom	United States
IAR, %	50	52	64	70	47	48
PER, %	50	48	36	30	53	52

### Diagnosis

Respondents' rhinitis was largely self-diagnosed in all countries surveyed, led by France (74%), followed by the United States (72%), Germany (62%), the United Kingdom (61%), Italy (60%), and Spain (50%). Spain reported the highest percentage of physician diagnoses (46%), followed by Italy (41%); only in France did less than one third of respondents receive a physician diagnosis. The percentage of respondents who were diagnosed by a friend or a family member varied from 7% in Italy and Spain to 18% in the United Kingdom and France. A negligible percentage was diagnosed by other means; that is, pharmacist, homeopath, physician's assistant, nurse practitioner, or information obtained in a physician's office or from an advertisement. (Respondents could provide more than 1 answer to this question.)

### Symptoms

**Figure 3:**
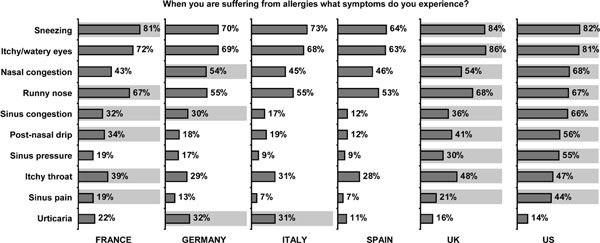
**Allergy symptoms experienced**.

A majority of respondents in all countries reported sneezing and itchy/watery eyes to be their most common symptoms (sneezing: 64%-84%; itchy/watery eyes: 63%-86%). Other symptoms elicited more varied responses. For example, 66% of respondents in the United States reported sinus congestion compared with only 12% of those in Spain (Figure 3).

There was a great amount of intercountry variability between the number of participants reporting nasal and sinus congestion and sinus pressure and pain. Nasal congestion was by far the most common symptom across countries. The percentage of positive responses to nasal congestion ranged from 43% in France to 68% in the United States.

**Figure 4:**
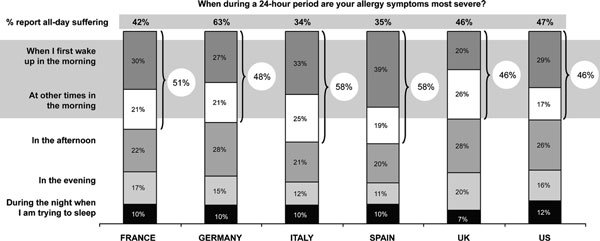
**Time of day when symptoms were most severe**.

Patients in all countries reported that their most severe symptoms occurred "when I first wake up in the morning" or "at other times in the morning." Fifty-eight percent of respondents in Spain and Italy identified the morning hours as the time they experienced their worst allergic symptoms, followed by France (51%), Germany (48%), and the United Kingdom and the United States (both 46%). Lesser percentages were noted for severe afternoon, evening, and nighttime allergies (Figure 4).

However, the nature of morning symptoms varied. Respondents in France and Spain identified sneezing as their most frequent symptom upon rising (60%), whereas in the United States it was nasal congestion (56%). More than half of Spanish respondents experienced itchy eyes and runny nose most frequently in the morning; Spain was the only country that reported a 50% or greater response rate for both of these symptoms.

Overall, US participants reported more negative emotional effects from morning allergies than those surveyed in the other 5 countries. In general, irritability and fatigue were the most common effects of symptoms, with some variation between the countries. Responders in Italy reported a higher degree of irritability (58%), followed by the United States (55%) and the United Kingdom (53%), compared with only 24% of responders in Spain. French participants noted the highest degree of fatigue (59%), followed by the United States (52%); it was identified as a primary morning concern by 31% to 44% of patients in the other countries.

**Figure 5:**
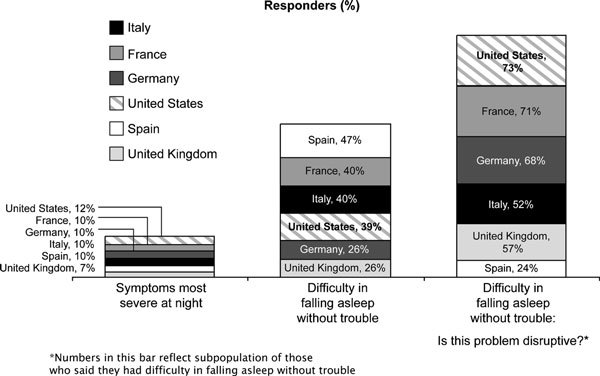
**Impact of allergies on sleep**.

A large percentage of responders reported difficulty in falling asleep without trouble, ranging from 47% in Spain to 26% in Germany and the United Kingdom. When respondents were asked whether this problem was disruptive, 73% in the United States, 71% in France, 68% in Germany, 57% in the United Kingdom, and 52% in Italy said that it was; however, only 24% of those in Spain (many of whom had reported difficulty in falling asleep) said that it was disruptive (Figure 5).

From a list of 12 topics on the impact of AR on lifestyle, breathing normally was ranked as the first and most common difficulty across all countries (Table 3). The topic ranked last in all countries was the impact on the ability to take care of children. Overall, issues relating to sleep and rest were rated as more difficult than issues relating to social interaction. There was considerable divergence about the ability to participate in outdoor activities.

**Table 3:** Ranking by Country of Lifestyle Impact of Allergies

Function or Activity	France	Germany	Italy	Spain	United Kingdom	United States
"When I'm suffering from allergies, I find it difficult to···"						
breathe normally	1	1	1	1	1	1
···get enough sleep to feel well rested in the morning	2	3	6	4	3	3
···fall asleep without trouble	3	5	3	2	5	5
···feel well rested during the day	4	4	4	3	7	2
···participate in outdoor activities	5	2	2	7	2	4
···take part in regular activities at home and at work	6	9	5	8	9	10
···sleep without snoring	7	8	11	5	10	8
···taste the food I eat	8	11	7	9	11	9
···keep my mood up and not feel unhappy or depressed	9	6	10	6	6	6
···think clearly	10	7	8	11	4	7
···take part in social activities with family and friends	11	10	9	10	8	11
···take care of my children	12	12	12	12	12	12

### Treatment regimens

Despite the large rate of reported self-diagnoses, in all countries the highest response to the question "Why did you first try the product you use most often?" was "Doctor recommended/prescribed it." This was true for both prescription (Rx) and over-the-counter (OTC) treatments, with the highest percentage of drug choices based on physician recommendation occurring in Spain (86% Rx; 76% OTC). Spain (66%) and France (62%) had the highest rates of Rx drug use, and the United Kingdom had the highest rate of OTC drug use (41%).

In the United Kingdom, 23% of responders selected their own medications regardless of physician input, and 28% of those in this subgroup chose OTC formulations. Friends and pharmacists were also identified as major influences in the selection of OTC formulations, but they had a considerably smaller influence on Rx drug choice. Advertising had some influence when it came to patient choice of OTC medications but was much less influential in Rx drug choice.

When questioned about their preferred form of allergy medication (Table 4), most respondents in the 5 European countries reported that they had tried tablets/pills/caplets, and of that group, a majority expressed preference for this form. In France, for example, 77% had taken tablets and 54% preferred them, followed by the United Kingdom with 75% reporting use and 53% expressing preference. Both German and Italian responders had used nasal sprays in larger percentages than tablets (71% and 52%, respectively), but neither group preferred nasal sprays to tablets. Twenty to 48% of respondents across countries also reported having tried eye drops. Much smaller percentages of responders had tried other vehicles of administration (eg, syrups, throat spray, liquid gels).

**Table 4:** Medications Used by Respondents (%)*

Form	France	Germany	Italy	Spain	United Kingdom
Tablets/pills/caplets	77/54	59/38	51/34	42/32	75/53
Nasal spray	53/12	71/35	52/25	30/20	56/21
Eye drops	41/18	48/10	42/14	20/9	43/12
Capsules	6/1	24/6	10/4	16/8	21/7
Syrups/liquids	18/3	13/1	11/4	12/7	15/3
Throat spray	24/5	12/2	19/8	16/8	14/2
Chewable	3/0	5/2	3/1	2/1	6/1
Quick-dissolve tabs	11/4	12/1	7/3	20/12	5/2
Liquid gels	1/1	13/6	6/4	5/2	2/0
Granules	26/12	4/0	3/1	2/1	1/0

*The first number is the percentage of respondents who have tried each form; the second number is the percentage of respondents who prefer that form.

Respondents from the 5 European countries answered questions designed to identify which brands (both prescription and OTC) they had used and which ones they had heard of and were likely to try in the future. As a rule in all countries, the brand that received the highest percentage of positive responses tended to achieve the highest degree of preference for current and future use.

Attributes listed by respondents of the ideal drug to treat allergies were fairly consistent across all countries. Efficacy, rapid onset of action, safety, and lack of side effects were rated as the most important attributes. Table 5 lists other desirable qualities identified by 80% or greater of allergy sufferers from the individual countries.

**Table 5:** Desirable Qualities of Allergy Medications

France	Germany	Italy	Spain*	United Kingdom
• "Puts me in control of my allergies"	• "Lets me eat, sleep, and breathe"	• "Relieves congestion and lets me breathe"	• "Puts me in control of my allergies"	• "Puts me in control of my allergies"
• "Doesn't make me drowsy"	• "Provides the right duration of relief"		• "Relieves congestion and lets me eat, sleep, and breathe"	• "Doesn't make me drowsy"
• "A leader in allergy relief"	• "Doesn't make me drowsy"		• "Lets me fall asleep"	• "Allows me to stay alert and focused"
• "Allows me to stay alert and focused"	• "Puts me in control of my allergies"		• "Lets me get a good night's sleep"	• "Relieves congestion and lets me eat, sleep, and breathe"
• "Lets me wake up symptom free"	• "Lets me wake up symptom free"		• "Doctor-recommended brand"	• "Provides full 24-hour relief"
• "Provides all-day relief into the next morning"			• "Provides the right duration of relief"	• "Lets me wake up symptom free"
			• "Can feel it working"	• "Works on multiple symptoms"
			• "Lets me taste my food"	

*No attribute was rated by more than 45% of respondents; attributes listed here had a 40% or greater response rate.

Ideal attributes of allergy medications change over time. In US respondents, attributes that became more significant were relief of symptoms, recommendation by physicians and pharmacists, freedom from sedating qualities and other adverse effects, and not interfering with a good night's sleep. Some remained unchanged, such as efficacy and safety, and others became less significant, including taste, reputation, and use by the whole family.

Although there are many treatment options for AR, it remains a largely undertreated condition. The percentage of nontreaters in this survey ranged from 11% in the United States to 21% in Germany. Reasons that respondents in this subgroup did not treat their AR included the following: delaying time to seek treatment because they believed that their symptoms were unimportant, finding the cost of medications too high, or having an inadequate relationship with their physician or health care provider [13]. Improving communication between clinicians and patients can lead to greater use of therapeutic interventions that will aid in symptom relief [13].

## Discussion

The information gathered from allergy sufferers through the present survey supports the observations of many clinicians that people with AR perceive their symptoms as causing significant QoL disruptions. Allergic rhinitis has an unquestionable impact on QoL, including limitations on daily activity and decreased productivity at work and school.

The information provided by QoL questionnaires focusing on AR has also made it clear that patients with AR are impaired in many domains of their daily lives [13]. Symptoms of AR are generally most prevalent in the morning, leading to problems throughout the day. In 2005, Long conducted a survey of 1000 adults with AR; 65% had symptoms upon awakening and 83% of respondents had symptoms throughout the morning [14]. Forty-nine percent of those surveyed said that their AR was at its worst in the morning. In the subgroup of those who experienced symptoms on awakening, 80% felt less energetic, 77% more fatigued, 75% less motivated, and 74% more irritable than usual [14]. In the present survey, participants reported that all symptoms were most severe when they first woke up in the morning or during other times in the morning.

Respondents in all countries said they had experienced difficulty in falling asleep. Sleep disturbances are commonly noted in patients with AR and contribute to a reduction in QoL [15]. One survey involving nearly 5000 subjects with AR found that those who frequently had symptoms at night were significantly more likely to report habitual snoring, chronic excessive daytime sleepiness, or chronic nonrestorative sleep (*P *< 0.0001) [16]. A smaller study comparing subjects with seasonal AR (SAR) with healthy volunteers found a statistically significant relation between nighttime sleep disruption and daytime sleepiness in those with SAR (*P = *0.048). However, the daytime sleepiness could be related to the SAR symptoms as well as the impaired sleep [17].

A recent 2-week study of 483 subjects with AR randomized to desloratadine 5 mg or placebo found that treatment with the second-generation antihistamine desloratadine significantly improved total RQLQ scores between baseline and day 14 (*P = *0.0003 vs placebo). Significant decreases in RQLQ subdomain scores were also seen with desloratadine [18]. In another 2-week study of subjects with SAR (N = 688), fexofenadine 120 mg therapy led to significant improvements in overall QoL, as measured by the RQLQ (*P *≤ 0.005 vs placebo) [19].

Given the pervasive adverse effects of AR on function and capacity, it is evident that medical treatment for AR should address issues beyond clinical outcome measures. One of the chief goals of therapy is to improve patients' well-being and their ability to function. Treatment modalities should not only provide optimum clinical benefits; they should also increase patient satisfaction [20,21]. The allergy treatment outcome that survey respondents in all countries desired most was the ability to breathe normally; getting restorative sleep was ranked second and feeling well rested during the day third. Attributes of the ideal AR medication identified by respondents in most countries included rapid onset of action, safety, and minimal bothersome side effects.

Responses to the survey suggest that AR is often perceived as causing daytime fatigue. It is possible that some people with AR could be experiencing the sedating effects of first-generation antihistamines in addition to the impairment and sleep disturbances caused by their symptoms.

Although not specifically addressed by the survey administrators, it should be noted that there are differences among the 5 European countries in allergenic pollens. Grass, birch, mugwort, and ragweed pollen predominate in central Europe and in mountainous areas, whereas olive, cypress, and plants in the genus *Parietaria *dominate in the Mediterranean region [22]. Although pollen seasons tend to be shorter in northern climates with colder weather, allergy severity varies from year to year depending on weather patterns, especially rainfall. Due to higher pollution levels, populations in more urbanized regions and countries will experience greater sensitivity to allergens [22]. These factors may help to explain the variation between countries seen in this survey and those recently reported in a broader range of countries [23].

## Conclusions

Results from this survey highlight the range of allergic symptoms that negatively impact the lifestyle of people with AR. Patients clearly want treatment options that improve their QoL and restore their ability to engage in activities of daily living.

## Note

Dr. Canonica and Dr. Mullol have received research grants from Schering-Plough Corporation. Dr. Mullol has participated in advisory boards and received honoraria from Schering-Plough Corporation. Dr. Pradalier has no conflicts of interest. Dr. Didier has received honoraria from Schering-Plough Corporation.
